# EML4-ALK testing in non-small cell carcinomas of the lung: a review with recommendations

**DOI:** 10.1007/s00428-012-1281-4

**Published:** 2012-07-24

**Authors:** Erik Thunnissen, Lukas Bubendorf, Manfred Dietel, Göran Elmberger, Keith Kerr, Fernando Lopez-Rios, Holger Moch, Wlodzimierz Olszewski, Patrick Pauwels, Frédérique Penault-Llorca, Giulio Rossi

**Affiliations:** 1 Department of Pathology, VU University Medical Centre, Amsterdam, The Netherlands; 2 Institute for Pathology, University Hospital Basel, Basel, Switzerland; 3 Institute for Pathology, Charité, Charité Campus Mitte, Berlin, Germany; 4 Department of Pathology, Solna, Karolinska University Hospital, Stockholm, Sweden; 5 Department of Pathology, Aberdeen University Medical School, Aberdeen, Scotland UK; 6 Laboratorio de Dianas Terapéuticas, Centro Integral Oncológico Clara Campal, Hospital Universitario Sanchinarro, Madrid, Spain; 7 Institute for Surgical Pathology, Universitätsspital Zurich, Zurich, Switzerland; 8 Department of Pathology, Maria Sklodowska-Curie Institute of Oncology, Warszawa, Poland; 9 Department of Pathology, Antwerp University Hospital, Edegem, Belgium; 10 Department of Pathology, Centre Jean Perrin, Clermont-Ferrand, France; 11 Sezione di Anatomia Patologica, Azienda Policlinico di Modena, Modena, Italy

**Keywords:** Anaplastic lymphoma kinase, Rearrangement, Crizotinib, Algorithm, Guidelines, Non-small cell lung cancer

## Abstract

In non-small cell lung cancer, epidermal growth factor receptor gene mutations and anaplastic lymphoma kinase (ALK) gene rearrangements have a major impact upon the level of response to treatment with specific tyrosine kinase inhibitors. This review describes the molecular basis of ALK inhibition, summarizes current data on the effectiveness and safety of ALK inhibition therapy, describes the different testing methodologies with their advantages and disadvantages, provides a suggested testing algorithm and puts forward a proposal for an external quality assessment program in ALK testing.

**Electronic supplementary material:**

The online version of this article (doi:10.1007/s00428-012-1281-4) contains supplementary material, which is available to authorized users.

## Introduction

In 2008, lung cancer was the most commonly diagnosed cancer and caused the highest number of cancer-related deaths [1]. Currently, only 10–15 % of lung cancer patients will be cured [2]. Strategies to maximize treatment benefit have centred on individualizing treatment according to the molecular profile of the disease [3, 4]. Mutations of the epidermal growth factor receptor (EGFR) gene have a major impact upon the level of response to treatment with tyrosine kinase inhibitors [5]. In addition to mutations in the EGFR gene, alterations in hepatocyte growth factor receptor (MET), vascular endothelial growth factor (VEGF), VEGF receptor, GTPase KRAS (KRAS), receptor tyrosine protein kinase erbB-2 (HER2), echinoderm microtubule-associated protein-like 4-anaplastic lymphoma kinase (EML4-ALK), phosphatidylinositol-4,5-bisphosphate 3-kinase catalytic subunit alpha isoform, serine/threonine protein kinase B-raf, insulin-like growth factor 1 receptor [6], ROS1 [7], and the KIF5B and RET fusion gene [8, 9] have also been implicated as oncogenic drivers in the pathogenesis of non-small cell lung cancer (NSCLC), especially adenocarcinoma.

In 2007, Soda et al. [10] reported that a small inversion within chromosome 2p results in the formation of a fusion gene comprising portions of the EML4 gene and ALK in NSCLC cells. A recently developed MET inhibitor (PF-02341066, crizotinib; Pfizer) was shown to also inhibit ALK [11, 12]. Phase I [13, 14] and II [15, 16] studies have revealed encouraging data [14], leading to an accelerated approval of crizotinib by the FDA in August 2011 [16]. Pathologists will play a key role in the identification of aberrations of ALK in NSCLC. The aim of this article is to provide information on how ALK alterations in NSCLC can be detected, with an emphasis on the uncertainties regarding methodology.

## Molecular basis of ALK inhibition therapy

**Fig. 1:**
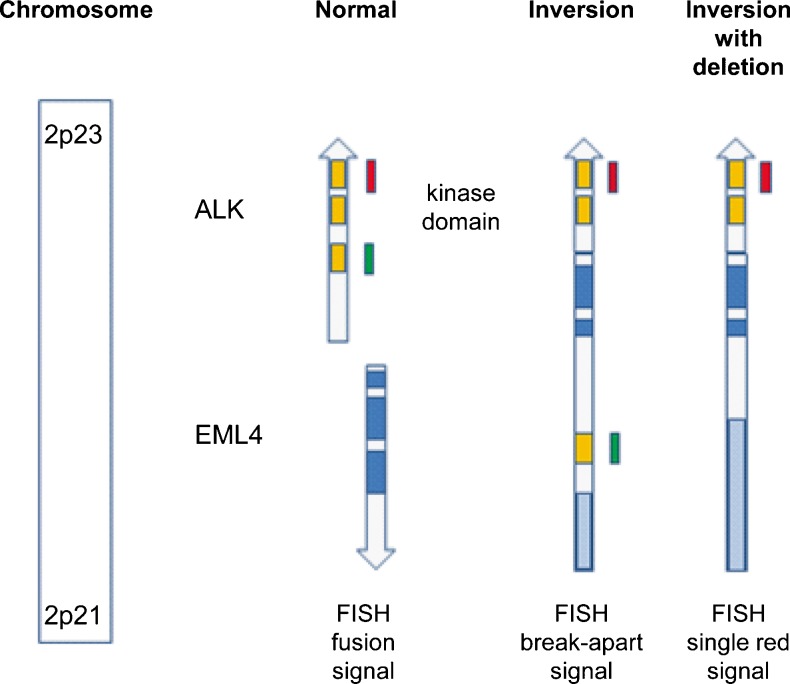
Signal pattern of Vysis ALK Break Apart FISH Kit. ALK and EML4 are located on chromosome 2p21–2p23. Note that EML4 is normally on the opposite strand to ALK, and that both probes on the ALK gene (*red orange* and *green*) are close together, explaining the FISH fusion signal. The EML4-ALK fusion gene is the result of an inversion of the N-terminal portion of EML4 with the kinase domain of ALK [10]. This inversion leads to an increased distance between the red orange and green probes. A deletion of the proximal part combined with the inversion explains the single red orange signal. The EML4-ALK fusion protein has a fully functional ALK kinase domain and has gain-of-function properties [5, 10]. Cells are considered ALK FISH positive when there is: (1) ≥1 set of red and green signals that are ≥2 signal diameters apart, or (2) a single red signal without a corresponding green signal in addition to fused (normal) signals. A sample is considered negative if <5 cells (<10 %) are positive and positive if >25 cells (>50 %) are positive. A sample is considered equivocal if 5–25 cells (10–50 %) are positive

Transforming rearrangements of the ALK gene were first identified in anaplastic large cell lymphomas [17, 18], and subsequently in inflammatory myofibroblastic tumours [19] and a subset of NSCLC [10]. An inversion event on the short arm of chromosome 2, resulting in the fusion of ALK gene with the EML4 gene locus, is the most common aberration of the ALK gene in lung cancer (Fig. 1) [10]. This rearrangement leads to the production of a chimeric protein, which has constitutive ALK kinase activity [14]. Over ten EML4-ALK variants have been identified in lung cancer so far, as well as other fusion partners TFG and KIF5B [20]. In addition, as single fluorescence in situ hybridization (FISH) signals (3′) are also associated with a positive response to crizotinib treatment, it seems that small deletions of 5′ end of the ALK gene may also occur [14, 21].

Current estimates suggest that the EML4-ALK fusion is present in approximately 3–6 % of adenocarcinomas, depending upon on the population studied and the ALK detection methods used [22, 23].

Clinical characteristics associated with the EML4-ALK gene fusion are adenocarcinoma histology, never/light smoking history and younger age [24–26]. However, these characteristics are not shared by all carriers. The ALK fusion has also been detected in older patients (aged 76 years) with a smoking history [24]. Therefore, clinical characteristics are insufficient and molecular testing is necessary to determine ALK status [27].

ALK-positive tumours have been detected in all histological subtypes of adenocarcinoma, but especially in solid signet-ring cell and mucinous cribriform patterns [24, 28–31].

## Effectivene*s*s and safety of ALK inhibition therapy

Crizotinib is the most advanced ALK inhibitor in clinical development [3]. In ALK-positive patients (*n* = 119), an overall response rate (complete responses + partial responses) of 61 % (95 % CI, 52–70 %), a clinical benefit rate (complete responses + partial responses + stable disease) of 88 % and a preliminary median progression-free survival of 10 months (95 % CI, 8–15 months) have been shown [13]. The median overall survival has not been reached. In a retrospective case-match analysis, Shaw et al. [32] found that ALK-positive NSCLC patients had a longer overall survival rate after crizotinib as second- or third-line therapy. Crizotinib seems to show poor penetration of the blood–brain barrier [33].

Crizotinib is well tolerated, with reported treatment-related adverse events of nausea (46 %), vision disorder (45 %), vomiting (39 %) and diarrhoea (29 %), mostly of grade 1/2. Grade 3/4 adverse events were reported in 15 % of patients (mostly increased alanine aminotransferase, dyspnoea and neutropenia) [13].

A proportion of ALK-positive patients with NSCLC (in most studies <10 %) continue to show progressive disease in spite of crizotinib treatment [15, 34]. Several mechanisms of resistance have been suggested to explain this lack of efficacy: (1) ALK kinase domain mutations (found in 4/11 samples); (2) copy number gain of the ALK gene rearrangement (found in 2/11 samples); (3) EGFR/KRAS mutations (found in 3/11 samples) [35]. Some patients never showed (intrinsic resistance) and some initially showed (acquired resistance) a response to treatment [35].

Neither percentage of positive cells nor signal copy number appears to be predictive of benefit from ALK inhibition treatment [21]. In other words, using ALK FISH, there is no greater likelihood of a treatment response to crizotinib in a patient with a sample showing nearly 100 % positivity than for a patient with a sample showing just above the positivity threshold. However, it is still important to know the percentage of positive cells to determine whether the threshold has been reached.

## Detection of ALK gene rearrangements

Increasing availability of targeted agents with selective biomarkers has introduced new challenges in NSCLC diagnosis [36]. Issues related to small sample diagnostics, the interaction between pulmonologists and pathologists, tissue management and required clinical information have recently been described [37]. Current diagnostic approaches to detect ALK rearrangements include immunohistochemistry (IHC), FISH and reverse transcriptase polymerase chain reaction (RT-PCR). Unpublished information from pilot studies suggests that ALK fusions may be demonstrated with massive parallel sequencing. However, sufficient details for an adequate comparison with FISH are currently lacking.

### Tissue management

With personalized tumour medicine in mind, tissue sample size should be maximized whenever feasible. In addition, tissue handling, processing and sectioning should be standardized to minimize wastage and optimize use of tissue for staining procedures and PCR-based molecular tests. Histological and cytological specimens are both potentially suitable for ALK testing. Currently, the only clinically validated test to determine ALK status is the Vysis/Abbott ALK FISH break apart test. However, this test and the interpretation algorithm are only designed and validated for histopathological use on intact formalin-fixed paraffin-embedded (FFPE) tissue biopsies or resection specimens. If cytological material is to be used, laboratories must take steps to validate their testing methodology.

Tumour tissue should preferentially be taken from metastases if access is less dangerous. Sampling of these sites ensures that the more important fraction of total tumour cell burden is collected. Furthermore, potential heterogeneity problems within the primary tumour are avoided when sampling metastases.

#### Standardized tissue and cell processing

Tissue handling steps have recently been described [37]. While so-called ‘reflex’ IHC and molecular testing provides a rapid diagnosis, it should be avoided if tissue resources are marginal. In anticipation of obligatory biomarker testing, an algorithm for reflex block cutting could be employed.

If the initial tissue sample is small, some (three to four) additional sections (extra spare cut sections) may be taken to avoid tissue loss by recutting. The quality of some epitopes and DNA/RNA may be lost (within hours/days) when sections are not immediately used [38–40]. Various treatments for the cut sections may prevent oxidation [37].

An algorithm that laboratory staff may find useful when handling lung biopsies is presented in Electronic supplementary material (ESM 1).

### Clinical diagnostic tests

#### IHC

Immunohistochemical detection of the ALK protein has considerable potential as a screening tool to test NSCLC samples for ALK rearrangements. The biological premise is that the translocation or inversion of part of the ALK gene with its numerous possible partners leads to over-expression of the ALK protein, and therefore overactivity of the ALK tyrosine kinase. This protein ALK tyrosine kinase is the target of crizotinib. It therefore makes sense to assess the drug target directly.

IHC has several advantages over either an ISH method (especially when based on fluorescent probes) or an approach based upon mRNA (RT-PCR) to detect the actual rearrangement. These advantages are that it is cheap, rapid and easily integrated into a diagnostic protocol and familiar to all pathologists. Although IHC is only minimally quantitative, in practice it is used as a semiquantitative test [41]. However, challenges with IHC are: (1) the tissue preparation; (2) the choice of antibody; (3) the signal enhancement system; (4) the scoring system.

##### Pre-analytical phase

Currently, the role that pre-analytical issues, like fixative and duration of fixation, may have on ALK epitope preservation has not been evaluated. However, compared to what has been observed for other antibodies [39], here the pre-analytical steps appear to be particularly critical and mistakes are irremediable. False-negative cases may be due to poor fixation. Such issues may be of particular concern in surgically resected tumours, where a gradient in positivity can be observed due to the gradient in fixative penetration in the sample [42]. Ultimately, any test for abnormal ALK genes in NSCLC is likely to be most important in small biopsy or cytology samples, where intra-sample heterogeneity is less likely to be appreciated because fewer cells are present, but where the possibility of heterogeneity of expression may lead to a false-negative test.

##### Analytical phase

One of the particular challenges in IHC detection is that the protein concentrations in ALK-rearranged NSCLC are relatively low. Standard detection methodology, as used in the identification of ALK-rearranged anaplastic lymphomas, is inadequate for the detection of all cases of ALK-rearranged NSCLC. Currently, there are three primary antibodies commonly referred to in the published literature; clone 5A4 (Novocastra, Leica, but also available pre-diluted from Abcam), ALK1 (Dako) and D5F3 (Cell Signalling Technology). Experience with the first two antibodies is widest, and more success (and excellent comparison with the outcome of FISH testing for the rearrangement) has resulted from their use with detection systems with a substantial degree of signal amplification (Leica/Novocastra Novolink, Dako Advance, Tyramide, Envision+, Ventana i-view). For more details, see Tables 1 and 2. If using FISH as the gold standard, use of these antibodies in this way has resulted in both a very high negative predictive value (all IHC-negative cases are also FISH-negative) and high positive predictive values (90–100 % probability of being FISH positive when IHC is strongly positive) [23, 24, 43–51]. For more details, see “Concordance between IHC and FISH” section. Good concordance has also been shown between RT-PCR and the antibodies ALK1 [22, 44] and 5A4 [45]. With current knowledge, all IHC positive (+/++/+++) cases would be best screened with FISH to confirm probable rearrangement.

**Table 1:** IHC protocols described in the literature

	Antibody	Source	HIER	Dilution	Incubation	Detection system
Yi et al. [46]	ALK1	Dako	EDTA, pH 8, 30 min	1/100	30 min at RT	Advance (Dako)
Yang et al. [43]	ALK1	Dako	EDTA, pH 8, 30 min	1/100	30 min at RT	Advance (Dako)
Paik et al. [47]	5A4	Abcam	CC1 (Ventana), 1 h	1/30	2 h at 42 °C	i-view (Ventana)
McLeer-Florin et al. [48]	5A4	Abcam	CC1 (Ventana), 1 h	1/50	2 h at 37 °C	i-view (Ventana)
Hofman et al. [45]	5A4	Abcam	pH 9, 40 min, 97 °C	1/50	30 min RT	EnVision Flex (Dako)
Kim et al. [27]	5A4	Novocastra	CC1 100 °C, 20 min	1/30	2 h at 42 °C	i-view (Ventana)
Mino-Kenudson et al. [49]	D5F3	CST	EDTA pH 8 pressure cooker	1/500^a^	Overnight	EnVision+ (Dako)
	D9E4	CST		1/100^b^		
	ALK1	Dako		1/50^a^		
				1/2^b^		

*HIER* heat-induced epitope retrieval, *EDTA* ethylenediaminetetraacetic acid, *RT* room temperature, *CST* cell signaling technology

^a^For anaplastic large cell lymphomas

^b^For lung adenocarcinomas and inflammatory myofibroblastic tumours

**Table 2:** IHC scoring systems

	Score			
	0	1+	2	3
Yi et al. [46]	No staining	Faint cytoplasmic staining	Moderate smooth cytoplasmic staining	Intense granular cytoplasmic staining in ≥10 % of tumour cells
Kim et al. [27]	No staining	Faint or weak staining intensity with >5 % tumour cells or any staining intensity with ≤5 % tumour cells ^a^	Moderate staining intensity with >5 % tumour cells ^b^	Strong and granular staining intensity with >5 % tumour cells ^c^

^a^Average of 14.7 % positively stained cells

^b^Average of 58.2 % positively stained cells

^c^Average of 97.3 % positively stained cells

In the literature (Table 1) and in the experience of the authors, there is some variation in the best concentrations of various primary antibodies and the antigen retrieval methodology. Personal experience with the 5A4 clone has found better results with pH6- when compared to pH9-buffered solution.

Comparing primary antibodies is very difficult since there are few published studies with comparative data. One study did compare the D5F3 clone with the ALK1 clone and found the former far superior [49], but the latter was not used with any special detection amplification. At this moment, it is very difficult to reach a conclusion from the literature; there may be a marginally better performance from the 5A4 clone when compared to the ALK1 clone, borne out by anecdotal experience, but it must be emphasized that other variations in study methodology make comparison problematic.

##### Post-analytical phase

In NSCLC, ALK-rearranged staining is cytoplasmic. It may have a granular character, and in some cases there may be membrane accentuation. Opinions differ as to the degree of staining heterogeneity that may be encountered. Whilst some assert that it is generally homogeneous in tumours, others, including the authors, have certainly seen variation in staining intensity in sections of surgically resected tumours (see comment above regarding fixation).

One of the most neglected issues in the literature is the question of definitions of degree of staining. Many studies do not even indicate how different grades of staining were defined. There is no clear consensus on this matter. Prospectively, it may be of value to determine a so-called histoscore (H-score) utilizing the proportion of tumour cells showing each different intensity (+, ++ and +++) of staining. Assessment of staining intensity is very subjective. The use of successive microscope objectives with related spatial resolutions as a physical aid in establishing intensity (as first applied on HER2 by Ruschoff et al. [50]) may lead to more uniformity in intensity scoring. Strong staining (+++) is clearly visible using a ×2 or ×4 objective, moderate staining (++) requires a ×10 or ×20 objective to be clearly seen, whilst weak staining (+) cannot be seen until a ×40 objective is used. This approach is called the modified H-score. Frequently used scoring systems are presented in Table 2.

For the time being, the modified H-score is recommended because it will allow sufficient detail for comparison with other studies. It may turn out that a more simplified scoring system is suitable as well. Ultimately, studies are required where prospectively determined IHC scores can be directly related to clinical outcomes in patients treated with crizotinib.

Pathologists should be aware of various artefacts that may lead to false-positive staining; these are generic issues, not necessarily specific to ALK IHC. The possibility of false-negative tests can be guarded against, at least to some extent, by the use of positive control material with every test staining run. Such material may not be readily available, and it is important that the control material is from ALK-rearranged NSCLC or similar and has the same levels of ALK protein epitopes. ALK-rearranged lymphomas are not suitable as a control because he protein epitope level is much higher than in NSCLC, thus giving false confidence of sufficient staining.

#### FISH

**Fig. 2:**
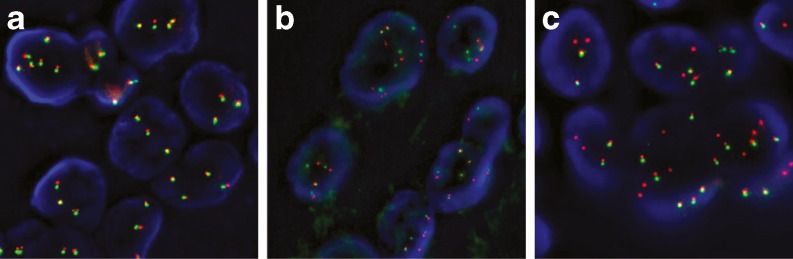
Representative examples of ALK FISH findings in three pulmonary adenocarcinomas (Vysis ALK Break Apart FISH probe). All three carcinomas show increased ALK copy number. **a** Normal signals, no rearrangement. Note that some of the signals are fused and produce a yellow signal, while others have *green* and *red* signals in close proximity. **b** One or two break apart signals per nucleus, indicative of inversion. **c** Single red signals, indicative of inversion and deletion. Note that the cancer cells in **b** and **c** contain both rearranged and normal ALK signals. Cells are considered ALK FISH positive when there is: (1) ≥1 set of red and green signals that are ≥2 signal diameters apart, or (2) a single red signal without a corresponding green signal in addition to fused (normal) signals. A sample is considered negative if <5 cells (<10 %) are positive and positive if >25 cells (>50 %) are positive. A sample is considered equivocal if 5–25 cells (10–50 %) are positive

The majority of clinical studies examining ALK mutations have used FISH. In the USA, prescription of crizotinib is dependent upon the use of the Vysis ALK Break Apart FISH Probe Kit (Abbott Molecular, Inc.) [16]. Although FISH can be performed on FFPE tumour specimens and detect multiple ALK fusion variants [28], there are various challenges related to the FISH technique, e.g. the break apart red and green signals indicating ALK rearrangements (Fig. 2) can be subtle and occasionally difficult to recognize.

##### Pre-analytical phase

A protocol for cytology is provided in ESM 2.

Processing and sectioning*—*For an effective procedure for surgical specimens, it is recommended that embedded tissues do not exceed 2 cm in length and 3 mm in thickness. Samples should be processed according to a standard protocol.

In practice, ALK testing works well on 3- to 5-μm sections. A minor preference exists for a thickness of 5 μm. Sections should be mounted on coated slides to prevent cleavage artefacts and washing off during the procedure. Sections should be dried at 60 °C for 1 h or at 45 °C overnight.

The maximum storage time for tissue or cell block sections should be 1–6 months (based on the authors' experience) and 6–12 months for conventional cytological specimens [52] to avoid hybridization failure and either false-negative or false-positive results. A protective coating of paraffin to histological sections prevents loss of antigenicity caused by photo-oxidation and drying of the tissues [37]. In order to avoid problems of irregular or background staining of histological specimens, it is essential to deparaffinize the samples properly [37].

Relocation of tumour cells by a software-driven automated stage is highly desirable for ‘restrained’ cytological specimens with a tumour cell proportion of <50 %. This allows precise distinction of the tumour cells from admixed benign cells and facilitates review of the results at the microscope.

##### Analytical phase

- Method—Use of diagnostic kits certified by the FDA (Abbott Vysis ALK Break Apart FISH Probe Kit, Abbott Molecular, Inc.) is recommended. The package insert contains a detailed protocol of the technical procedures and interpretation. In practice, the Vysis kit has some limitations: (1) appropriate optical filters are required for the probes used; (2) the green signal may fade earlier than the red signal, increasing the likelihood of false-positive single-red signals. Other, not yet FDA-approved, FISH assays are commercially available (e.g. ZytoLight® SPEC ALK/EML4 TriCheck™ Probe, ZytoVision). Every assay should undergo validation in the laboratory before clinical implementation. This validation can be conducted with five to ten positive and five to ten negative cases. The results are compared with a reference centre, and concordance of at least 95 % should be obtained. In addition, laboratories should participate in external quality assessment schemes as soon as they become available. When a laboratory authorized to conduct FISH adopts another bright-field ISH for diagnostic purposes, an internal validation can be conducted in the laboratory comparing the new technique with FISH. Again, a concordance of 95 % must be obtained. This 95 % threshold may change in time, after more experience is gained with the external quality assessment. The use of standardized kits requires strict adherence to the manufacturers' instructions, i.e. without any deviation.
  A minimum norm for ALK experience is not known yet, but for Her2 it was suggested that at least 150 ISH cases per annum (minimum of 100 breast and 50 gastric cases by ISH per annum) would be the optimal volume to ensure quality and cost effectiveness [53] (see “Proposal for an external quality assessment program” section).
  The number of annual tests considered optimal for guaranteeing the technical sufficiency of a laboratory is 150 ISH tests on any type of tumour [51]. Analysis of complete histological sections is recommended in order to minimize signal loss on the Z-axis. In FISH procedures, it is essential to use fluorescence filters with excitation and emission wavelengths appropriate to the fluorochromes of the probes contained in the kit.
- Controls—With ISH techniques, the case for study serves as a control when consistently presenting signals, both in tumour cells and in the accompanying normal cells. Nevertheless, the use of separate controls is strongly advised. A pre-hybridization assessment of digestion is useful in difficult samples (e.g. very small biopsies with low tumour content).

##### Post-analytical phase

- Assessment of the results—A pathologist should interpret the results. When other persons score the specimens, they should be well trained and experienced in histo- and cytomorphology. In such a case, a pathologist should coordinate, validate, review and sign off the interpretation. A total of 50 nuclei should be scored using an epifluorescence microscope equipped with a DAPI, a Spectrum Orange, Spectrum Green and double-filter set, using a ×60–100 oil immersion objective lens. The spectral orange probe is mostly called the ‘red probe/signal’ in the literature, and this terminology has been used throughout the manuscript.
  Assessment must be conducted exclusively on nuclei with sufficient hybridization quality, in a consecutive manner, and with the microscope focus adjusted to each nucleus in order to correctly identify all the signals present in the nucleus of the cells.
  When conducting FISH as an ISH technique, given the difficulty of dark-field assessment, the following are recommended:
  - Cells are considered ALK FISH positive when: (1) at least one set of red and green signals have a distance between the signal borders of ≥2 diameters of the largest of the two signals (break apart of the two differently coloured probes indicates gene fusion by inversion), or (2) when there is a single red signal without a corresponding green signal in addition to fused (normal) signals (indicating gene fusion by inversion and deletion) (Figs. 1 and 2). Note that a single green signal without a corresponding red signal is considered negative. Similarly, an increased copy number of non-rearranged ALK genes with fused signals corresponds to polysomy of chromosome 2 or ALK amplification, but is negative for rearrangement.
  - A sample is considered negative if <5 cells (<10 %) are positive and positive if >25 cells (>50 %) are positive.
  - A sample is considered equivocal if 5–25 cells (10–50 %) are positive. In this case, a second reader should evaluate the slide. If the average of the two readings contains at least 15 % positive cells, the sample is considered ALK FISH positive. It should be kept in mind that the digestion time needs to be adjusted for some samples. Examples of typical FISH findings are shown in Fig. 2.
  During interpretation, the spatial distribution of the distance between the two signals in the *Z*-direction also needs to be taken into account. In cases of only one signal, adjusting the plane of focus (by turning the microscrew) is frequently necessary when using the conventional FISH microscope. An advantage of a digitized automatic FISH analysis system is that fluorescent images may be compressed in the *Z*-direction (*Z*-stacks), often making this automated stage more convenient than live microscopy. The presence of all signals in one horizontal plane facilitates scoring and review, and allows permanent documentation. In our experience, a positive scoring result that cannot convincingly be illustrated by fluorescence photography is of questionable value.
  Cytological specimens offer the advantage of analysing entire cells/nuclei containing all chromosomal signals. Instead, a part of the nuclei in histological tissue sections are truncated, leading to a lower number of fluorescence signals, and this effect depends on the section thickness.
  Note that in most ALK-positive samples, not all alleles are abnormal (e.g. only one in a disomic cell or one or two in tetrasomic cells). Typically, there is one predominant type of rearrangement in one sample (either break apart or deletion). Great variability in signal patterns within one sample might suggest technical artefacts.
  There is evidence that ALK rearrangements are truly diffuse rather than heterogeneous within ALK-positive tumours [21, 54]. First, IHC typically shows diffuse ALK expression in ALK-positive tumours [47, 55]. Second, the distribution of cell positivity by FISH is diffuse; there are no discrete foci of ALK rearrangements within a tumour [21, 54]. This suggests that any observed variability is not biological, but rather caused by technical factors.
  Given the presumed homogeneous distribution of ALK rearrangements, the percentage of FISH-positive cells in an ALK-positive tumour lying between 20 and 80 %, and not reaching 100 % may appear contrary. The ‘single red’ signal is found in a higher fraction of tumour cells than the ‘break apart’ signal [21]. This discrepancy can readily be explained by the distribution of signals in three-dimensional space and their projection by the assessing pathologist to the horizontal plane. A separated signal can be located anywhere on a virtual sphere around the other signal. In a disomic tumour cell with one rearranged ALK gene copy, the probability of detecting break apart signals in the two-dimensional projection may therefore not exceed 25–30 %.
  In the authors' experience, the minimum number of tumor cells required in histological specimens is at least 100, as approximately half will not be stained with the fluorescence probes for reasons related to the stereological analysis. Less than 40 tumor cells/neoplastic nuclei with FISH signals are unreliable for determining positivity.
  Increased ALK gene copy number (3 to >6 gene copies), which is mostly due to polysomy of chromosome 2, has been reported in 80 % of NSCLC [56]. In cases of polysomy, one rearranged signal per nucleus is sufficient for a ‘positive’. High gene copy number of non-rearranged ALK does not appear to influence response to crizotinib. A possible predictive value of the copy number of the rearranged ALK gene in tumour cells and its influence on the level of ALK protein expression remains to be investigated.
  Overall, interpretation of the ALK FISH analysis is more complex than for other FISH assays, e.g. with amplifications or interchromosomal translocation. This is due to the fact that: (1) fusion inversion occurs on the same chromosome arm—in contrast to fusion processes in SYT-SSX and BCR-ABL FISH tests, for example, where genes from different chromosomes are involved; (2) in some ALK wild-type cells (especially with larger nuclei or after prolonged digestion) the red and green signals may be slightly separated and the fusion to yellow fluorescence is not apparent; (3) the process of cutting and subsequent signal separation may lead to a low number of false-positive break apart signals in cells without the ALK fusion. These characteristics mean that a low threshold of positive cells can still select patients who will react to ALK inhibitor treatment. However, this complexity highlights the need for specific training and experience with ALK FISH.
- Personnel—The number of laboratory technicians who conduct tests and the number of pathologists who interpret them must be as low as possible in order to guarantee the effectiveness of the undertaking. Both the technicians and the pathologists must have undergone training. For assessing the post-analytical (interpretation) phase, correlation with the reference result of at least 95 % in 10–20 cases is recommended. Periodically, the aforesaid training should be refreshed in dedicated working sessions.
- Report—While the report should be adapted to the different information systems used in different hospitals, it must include, at minimum, the data presented in Table 3. The recommended turnaround time is <7 working days.

**Table 3:** Recommended data in a report on ALK testing by in situ hybridization

Clinical information
Patient identification
Identification of doctor making request
Dates of request and test
Identification of sample (case and block number)
Type of sample^a^
Anatomical origin
Gross specimen handling/molecular method
Date block received by laboratory
Block used for analysis
Probe used (supplier, approval by FDA or other agency)
Method of assessment (semi-quantitative, image analysis)
Threshold for positivity
Microscopy/molecular results
Number of nuclei assessed
Results
Positive (%)
Presence or absence of patterns indicating rearrangement (specify whether split or single)
Negative (% of translocated cells, if any)
Presence or absence of patterns indicating rearrangement
Inconclusive: Explanations for inconclusive answers^b^
Conclusion
Molecular test outcome and interpretation
Rearrangement associated with sensitivity
Name and signature of the pathologist(s) responsible for the investigation

^a^For example, bronchial–transbronchial biopsy, surgical specimen, core needle biopsy, transbronchial fine needle aspiration, effusion cytology or other

^b^For example, possible false-negatives due to low fraction of tumour cells or uncertain fixation time

FISH is not feasible in all laboratories and the results are not always clear. McLeer-Florin et al. [48] reported that 19 % of cases analyzed by FISH were not interpretable.

#### Concordance between IHC and FISH

The results from the three larger FISH/IHC concordance studies have shown that in FISH-negative cases, IHC is always negative (i.e. 100 % concordance) [46, 47, 57]. This complete concordance is also seen between a positive FISH result and an IHC 3+ score [46, 47, 57]. Very good concordance levels between FISH and IHC have also been demonstrated by Jokoji et al. [58]. McLeer-Florin et al. [48] also found good concordance between FISH and IHC. In their research, the Novocastra 5A4 antibody was used. The authors suggested using IHC to evaluate the cases that were not interpretable by FISH.

Some of the panel have had different experiences: namely that it is possible to have a sample that is IHC 3+ and FISH negative. In one such patient, a response to Crizotinib was seen (E.T.). Also a sample may be FISH positive and IHC negative. Some discordant results have been observed in tumours expressing ALK with a weak (1+) to moderate (2+) signal. Table 4 provides a summary of published articles comparing ALK analysis by FISH and IHC.

**Table 4:** Summary of published articles comparing ALK analysis by FISH and IHC

Reference	Antibody	Source	Detection method	Samples, *n*	ALK+		ALK−		Comments
					FISH	IHC	FISH	IHC	
Shaw et al. [25]	ALK1	Dako	n.a.	NSCLC, 141	19 (13 %)	19 (13 %)	n.a.	n.a.	Only FISH+ cases were confirmed with IHC
Boland et al. [44]	ALK1	Dako	n.a.	NSCLC, 335	6 (1.8 %)	6 (1.8 %)	n.a.	n.a.	The 6 cases testing positive using IHC were positive with FISH. 8 of the IHC ALK− cases were also tested using FISH. All were negative
Rodig et al. [24]	ALK1	Dako	EnVision+	Adeno, 358	20 (5.6 %)	n.a.	n.a.	n.a.	8 of the 10 FISH ALK+ cases with sufficient tissue were also IHC ALK+ (sensitivity = 80 %); 1 case ALK– by FISH was ALK+ by IHC
Mino-Kenudson et al. [49]	ALK1	Dako	EnVision+	Adeno, 153	22 (14.3 %)	n.a.	n.a.	n.a.	Sensitivity and specificity of IHC were 67 and 97 %, respectively
	D5F3	Cell ST	EnVision+	Adeno, 153	22 (14.3 %)	n.a.	n.a.	n.a.	Sensitivity and specificity of IHC were 100 and 99 %, respectively
Yi et al. [46]	ALK1	Dako	Advance	Adeno, 101	10 (9.9 %)	11^a^ (10.9 %)	91 (90.1 %)	90^a^ (89.1 %)	Of the 10 FISH+ cases, 8 were IHC 3+, 1 was IHC 2+ and 1 was IHC +; of the 91 FISH– cases, 2 were IHC 2+, 20 were IHC 1+, 69 were IHC 0; sensitivity and specificity^a^ of IHC were 90 and 97.8 %, respectively
Paik et al. [47]	5A4	Novocastra	i-view	NSCLC, 453	19 (4.2 %)^a^	26 (5.7 %)	434 (95.8 %)	427^a^ (94.3 %)	Of the 19 FISH+ cases, 16 were IHC 3+, 3 were IHC 2+; of the 434 FISH− cases, 7 were IHC 2+, 14 were IHC 1+, 413 were IHC 0; sensitivity and specificity^a^ of IHC were 100 and 92.5 %, respectively
Mitsudomi et al. [64]	5A4	Santa Cruz	EnVision+	NSCLC, 345	n.a.	12 (3.5 %)	n.a.	n.a.	All these 12 IHC ALK+ cases were also FISH ALK+; all IHC ALK– cases were also FISH ALK–
Martinez et al. [65]	D5F3	Cell ST	n.a.	NSCLC, 71	6 (8.5 %)	n.a.	n.a.	n.a.	All FISH ALK– negative cases were also IHC ALK–. 4/6 FISH ALK+ cases were also ALK IHC+; 1 case was IHC ALK–; 1 sample could not be analysed.
Paik et al. [66]	5A4	Novocastra	n.a.	NSCLC, 735	28 (3.8 %)	35 (4.8 %)	707 (96.2 %)	700 (95.2 %)	Of all cases, 15 were IHC 3+, 20 were IHC 2+, 20 IHC 1+, 700 were IHC0; all IHC0/1+ were ALK FISH–, all IHC 3+ cases were ALK FISH+, and 13 of the 20 IHC 2+ were FISH+; sensitivity and specificity^a^ of IHC were 100 and 96.2 %, respectively
				Adeno, 395	27 (6.8 %)	n.a.	368 (93.2 %)	n.a.	
McLeer-Florin et al. [48]	5A4	Abcam	i-view	Adeno, 441	n.a.	29 (6.5 %)	n.a.	n.a.	Of 81 cases with interpretable IHC and FISH results, 21 were FISH ALK+; of these 21 cases, 19 were IHC ALK+ and 2 were IHC doubtfully positive; of the 60 FISH ALK– cases, 1 was IHC ALK+ and 59 were IHC ALK–
Yang et al. [43]	ALK1	Dako	Advance	Adeno, 300	22/216 (10.2 %)	32 (10.7 %)^a^	194/216 (89.8 %)	268 (89.3 %)	All IHC 0 cases were FISH ALK– and all IHC 3+ were FISH ALK+; 96.9 % of IHC 1+ cases were FISH ALK− and 85.7 % of IHC 2+ cases were FISH ALK+

IHC scores of 0 and 1+ are regarded as negative; scores of 2+ or 3+ as positive

*Adeno* adenocarcinomas, *ALK* anaplastic large cell kinase, *FISH* fluorescence in situ hybridization, *IHC* immunohistochemistry, *n.a.* not available, *NSCLC* non-small cell lung cancer, *ALK+/–* positive/negative results for ALK rearrangement

^a^Using FISH as the gold standard

#### PCR-based molecular assays

RT-PCR provides a highly sensitive technique in which a very low copy number of RNA molecules can be detected. As for EML4/ALK the forward and backward primers are complementary to gene fragments, which are normally on opposite strands, the specificity is high. RT-PCR can be used on mRNA/cDNA to directly detect EML4-ALK; hence, it does not suffer from the problems inherent in interpreting FISH or IHC. Therefore, it has been used as a gold standard to assess the sensitivity and specificity of IHC [55], FISH [55, 59] and CISH [27, 59]. It has also been used as a stand-alone test instead of FISH or IHC [60].

However, RT-PCR has several disadvantages that make it unlikely to become the standard test for this mutation. Firstly, good quality RNA is required [59]: some of the amplicons are more than 1,000 bp in size and require proper cryopreservation of tumour samples that may be lacking in routine practice [55]. In FFPE sections, RT-PCR above 300 bp is not reliable. Secondly, multiplex systems are required because of the wide variations in fusion types [59]. Thirdly, only known alterations can be tested (at least 10 are currently known for ALK) [20]. Furthermore, the clinical relevance of multiplex RT-PCR is unclear because so far in all clinical trials FISH has been used to identity ALK-positive patients. RT-PCR is included in the diagnostic algorithm proposed by Just et al. [55], but only after IHC and FISH have been performed, and only as a source of further information on the ALK fusion variant and expression level, rather than for diagnosis.

RT-PCR for ALK mutation testing is being offered by some commercial vendors (e.g. Response Genetics, Inc., in the USA); however, it is not clear how reliable these tests are. The panel strongly suggested that any such tests should involve pathologists early in the development and should be diagnostically orientated.

## Testing algorithm

In the National Consensus from Spain [61], ALK rearrangement testing is recommended in patients with advanced NSCLC who are negative for the EGFR mutation (all histological subtypes in non-smokers; non-squamous-cell carcinoma subtype in current or ex-smokers). In contrast, the most recent NCCN guidelines (version 1.2012) [62] recommend ALK rearrangement testing concurrent with EGFR mutation testing for adenocarcinomas, large cell carcinomas and NSCLC NOS. This differs from recent guidelines in Switzerland [63] and France (French National Cancer Institute; INCa) [48] proposing ALK testing only by FISH and only in EGFR-negative KRAS-negative adenocarcinoma patients.

Since phase II and III clinical trials with crizotinib in ALK-positive patients have used FISH, this technique should be considered the ‘gold standard’ for determining ALK positivity. However, following successful validation with large series and different antibodies, IHC could also become a good screening method.

**Fig. 3:**
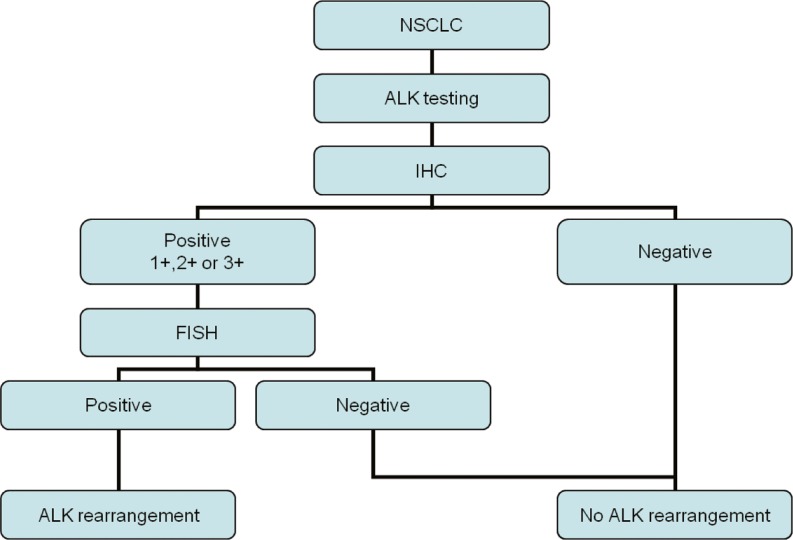
Possible ALK testing algorithm in NSCLC if IHC becomes fully validated

The authors agree that more data for an evidence-based algorithm are needed. It is possible that the algorithm presented in Fig. 3 may in the future be the algorithm of choice.

## Proposal for an external quality assessment program

For optimal ALK mutation testing in NSCLC, the quality of the sample, the analytical procedure and the reporting of the test result are crucial (Table 3). European quality assessment projects will examine different steps of ALK mutation testing.

One important ongoing quality assurance project in Europe is the FALKE (Fusion of EML4-ALK epidemiology Evaluation) project. This initiative was set up by the German Society of Pathology for German-speaking countries. The basis of this ring trial is to test 1,000 samples from NSCLC patients. For the FALKE project, it was decided to test all NSCLC and not to focus on adenocarcinoma. For all samples, ALK IHC and ALK FISH will be performed.

In 2012, the European Society of Pathology (ESP) will run two external quality assessment programs for lung cancer. Registration will take place through the ESP website (QA activities: http://esp-pathology.org/).

In the first round, starting March 2012, ALK FISH and optional ALK IHC will be offered. The pre-validated tissue microarray slides will have both positive and negative controls (n ≥10). The second round, in September 2012, will offer assessment of ALK1, EGFR and KRAS on tissue microarray samples. Participation is voluntary. Laboratories meeting the predefined performance threshold will be listed on the ESP website.

## Conclusions

For the personalized treatment of patients with NSCLC, it is necessary to sample as much tumour tissue as possible. Adequate clinical information is essential for the optimal tissue management.

Patients with ALK fusion adenocarcinomas confirmed by FISH have been shown to respond favourably to crizotinib treatment, and a phase III trial is currently ongoing. However, the question of how best to select patients that may benefit from crizotinib treatment remains to be answered.

There is evidence that IHC could be useful and less expensive than FISH. Some of the difficulties in interpreting FISH results may be alleviated by automated procedures.

Whether FISH or IHC becomes the primary method of ALK mutation testing in Europe, it is clear that rigorous quality assessment is of the utmost importance to ensure reliable results and appropriate patient selection.

## Electronic supplementary material

Below is the link to the electronic supplementary material.

ESM 1 (PDF 133 kb)

ESM 2 (PDF 90 kb)
