# Supplementary material for: EML4-ALK testing in non-small cell carcinomas of the lung: a review with recommendations
Source: Virchows Arch. 2012 Jul 24;461(3):245–57. doi: 10.1007/s00428-012-1281-4 (PMC3432214; doi:10.1007/s00428-012-1281-4)
Supplement: Supplementary file 1 — (PDF 133 kb) [file 428_2012_1281_MOESM1_ESM.pdf]

Online Resource 1

Algorithm for Laboratory Staff

This is an example of work-up instructions that have been introduced for lung biopsies in the institutions of three of the authors (M. Dietel, H. Moch and G. Elmberger).

- The material should be processed only by specially trained technicians in a dedicated laboratory, separate from the routine procedure.
- The embedding should be done under a stereo microscope to achieve optimal orientation of the small tissue fragments, e.g. same level of all particles in the paraffin block. Preferentially, core biopsies or forceps biopsies should be individually blocked.
- Trimming should be done carefully.
- Cutting of 3- to 5-µm sections should be performed with a rotation microtome in a quiet environment. Microtomes with a 'waterfall' bridge (section transfer system, Thermo Scientific, Walldorf, Germany) efficiently capture every tissue section and thus minimizes tissue loss.
- A 'touch and go' principle could be applied to make a first section to check for tumour presence and to verify NSCLC histology.
- Alternatively up-front reflex cutting of 10–12 sections for routine stains, mucin histochemistry, IHC and ISH could be prepared and stored while H&E is analyzed.
- PAS-D, TTF-1, p63 and CK5/6 are the minimal optional stains when NSCLC have been confirmed in routine stained histological material. Double stains are preferentially utilized in further IHC. Concerning cytological specimens, due to the limited number of slides, TTF1, p63 and mucin stain, in addition to H&E, are recommended to specify the histological type of carcinoma
- Finally, the last special staining section is notated or an additional H&E is cut to evaluate the remaining amount of tumour material still in the block. It can be very useful to inspect not only the last section cut, but also the remaining block, in order to avoid unpleasant surprises or being unnecessarily restrictive when ordering ancillary molecular tests.

If these regulations are followed carefully, in the majority of cases there will be enough leftover material to perform additional molecular analyses, if later requested by a clinician or by reflex testing ordered by the pathologist when diagnosing a primary NSCLC. The total number of tests that can be performed is determined by the size and number of the biopsies. 'Up-front' reflex (molecular) testing is optimal since it can both save tissue, if carefully planned and shorten turn-around time to the point that molecular results can be available at a multidisciplinary treatment conference. However, budgetary restrictions may interfere. Parallel testing of all relevant predictive molecular markers can actually save time and tissue since repetitive cutting and trimming is avoided and sometimes extraction protocols can allow for simultaneous extraction of both DNA (KRAS and EGFFR) and RNA (ALK).
