# Supplementary material for: EML4-ALK testing in non-small cell carcinomas of the lung: a review with recommendations
Source: Virchows Arch. 2012 Jul 24;461(3):245–57. doi: 10.1007/s00428-012-1281-4 (PMC3432214; doi:10.1007/s00428-012-1281-4)
Supplement: Supplementary file 2 — (PDF 90 kb) [file 428_2012_1281_MOESM2_ESM.pdf]

Online Resource 2

Basel (Switzerland) protocol for FISH on cytological specimens (Papanicolaou-stained smears or cytospins); Vysis ALK break-apart assay

Day 1:

- Place the uncovered slide(s) for 2 minutes in 2xSSC at 73°C in a microwave
- Place slide(s) for 30 minutes in 0.5mg/lm Pepsin in 0.01N HCL at 37°C in a microwave
- Place slide(s) for 5 minutes in formalin 1% / 100X $MgCl_2$ (25ml 10% formalin, 74ml PBS and 1ml 2M $MgCl_2$) at room temperature (RT)
- Place slide(s) for 5 minutes in PBS at RT
- Place slide(s) for 1 minute in 70% ethanol at RT
- Place slide(s) for 1 minute in 80% ethanol at RT
- Place slide(s) for 1 minute in 100% ethanol at RT
- Let dry slide(s) for 10 minutes at RT
- Warm up heating plate to 45°C
- Place slide(s) for 2 minutes on the heating plate
- Apply 10µl ALK probe "ready to use" to the target area of the slide
- Cover area with coverslip (20x20mm)
- Seal slide(s) with rubber cement or parafilm
- Place slide(s) on the surface of the HYBrite™ (or ThermoBrite™) denaturation/hybridization system. Fill in empty slots with blank glass slides
- Close the lid of the HYBrite™ and start the following program:
    - Co-denaturation: 8 minutes at 74°C
    - Hybridization: overnight at 37°C

Day 2:

- Warm up heating plate to 37°C
- Heat up water bath to 73°C together with a coplin jar containing 2xSSC/0.3% NP40 wash solution
- Remove the rubber cement or parafilm and the coverslip from the slide(s)
- Place slide(s) in the 2x SSC/0.3% NP40 at 73°C. When all slides are in the coplin jar incubate for 2 minutes

- Remove the slide(s) from the wash solution and place them in a coplin jar containing 2x SSC/0.1% NP40 and incubate the slides for 1 minute at RT
- Remove the slide(s) from the wash solution and place them vertically in the dark on a paper towel for drying
- Apply 10µl DAPI I counterstain onto the target area and place a 24x32mm coverslip over the DAPI solution, avoiding air bubbles
